# Supplementary material for: EnzML: multi-label prediction of enzyme classes using InterPro signatures
Source: BMC Bioinformatics. 2012 Apr 25;13:61. doi: 10.1186/1471-2105-13-61 (PMC3483700; doi:10.1186/1471-2105-13-61)
Supplement: Addtional file 5 — The Java code to format the data files, evaluate and predict. The file enzml_java_code.tar.gz contains the Java code used to format database data to ARFF and XML formats, to execute cross and train-test (jackknife) evaluations and to record evaluation results to database. More information is included in the readme.txt file and the Javadoc files. The code can be used with a MySQL database. To use a different database software, other JDBC drivers might be required. [file 1471-2105-13-61-S5.gz › java_code/enzml2011/doc/uk/ac/ed/inf/taweka/mulan/learn/ResultsOutputter.html]

ResultsOutputter


---


|  |  |  |
| --- | --- | --- |
| **Overview** \| **Package** \| **Class** \| **Use** \| **Tree** \| **Deprecated** \| **Index** \| **Help** | |  |
| **PREV CLASS**   NEXT CLASS | **FRAMES**    **NO FRAMES**     **All Classes** |  |
| SUMMARY: NESTED \| FIELD \| CONSTR \| METHOD | DETAIL: FIELD \| CONSTR \| METHOD |  |


---


## uk.ac.ed.inf.taweka.mulan.learn Class ResultsOutputter

```
java.lang.Object
  uk.ac.ed.inf.taweka.mulan.learn.ResultsOutputter
```

---

``` public class ResultsOutputter extends java.lang.Object ```

Class

**Version:**
:   15 Aug 2010

**Author:**
:   Luna De Ferrari luna.deferrari-at-ed.ac.uk

---

**Constructor Summary**

`ResultsOutputter()`


| **Method Summary** | |
| --- | --- |
| `uk.ac.ed.inf.utils.database.TableRow` | `addEvaluationResultsToTableRow(uk.ac.ed.inf.utils.database.TableRow row, mulan.evaluation.Evaluation results)`             adds the evaluation results to a table row object |
| `uk.ac.ed.inf.utils.database.TableRow` | `createEvaluationRecord(int i)`             Add timestamps and identifiers to the cross evaluation results |
| `mulan.evaluation.Evaluation[]` | `getEvaluationResults()` |
| `mulan.evaluation.Evaluation[]` | `getResults()` |

**Methods inherited from class java.lang.Object**

`equals, getClass, hashCode, notify, notifyAll, toString, wait, wait, wait`

**Constructor Detail**

### ResultsOutputter

```
public ResultsOutputter()
```


**Method Detail**

### addEvaluationResultsToTableRow

```
public uk.ac.ed.inf.utils.database.TableRow addEvaluationResultsToTableRow(uk.ac.ed.inf.utils.database.TableRow row,
                                                                           mulan.evaluation.Evaluation results)
```

:   adds the evaluation results to a table row object

---


### createEvaluationRecord

```
public uk.ac.ed.inf.utils.database.TableRow createEvaluationRecord(int i)
```

:   Add timestamps and identifiers to the cross evaluation results

    :   **Parameters:**: `i` - the index of the cross evaluation results record (the fold) **Returns:**: a record row

---


### getResults

```
public mulan.evaluation.Evaluation[] getResults()
```

---


### getEvaluationResults

```
public mulan.evaluation.Evaluation[] getEvaluationResults()
```


---


|  |  |  |
| --- | --- | --- |
| **Overview** \| **Package** \| **Class** \| **Use** \| **Tree** \| **Deprecated** \| **Index** \| **Help** | |  |
| **PREV CLASS**   NEXT CLASS | **FRAMES**    **NO FRAMES**     **All Classes** |  |
| SUMMARY: NESTED \| FIELD \| CONSTR \| METHOD | DETAIL: FIELD \| CONSTR \| METHOD |  |


---
